# Cannabinoids and Endocannabinoid System Changes in Intestinal Inflammation and Colorectal Cancer

**DOI:** 10.3390/cancers13174353

**Published:** 2021-08-28

**Authors:** Viktoriia Cherkasova, Olga Kovalchuk, Igor Kovalchuk

**Affiliations:** Department of Biological Sciences, University of Lethbridge, Lethbridge, AB T1K 7X8, Canada; viktoriia.cherkasova@uleth.ca

**Keywords:** cannabinoids, intestinal inflammation, colorectal cancer

## Abstract

**Simple Summary:**

In recent years, multiple preclinical studies have shown that changes in endocannabinoid system signaling may have various effects on intestinal inflammation and colorectal cancer. However, not all tumors can respond to cannabinoid therapy in the same manner. Given that colorectal cancer is a heterogeneous disease with different genomic landscapes, experiments with cannabinoids should involve different molecular subtypes, emerging mutations, and various stages of the disease. We hope that this review can help researchers form a comprehensive understanding of cannabinoid interactions in colorectal cancer and intestinal bowel diseases. We believe that selecting a particular experimental model based on the disease’s genetic landscape is a crucial step in the drug discovery, which eventually may tremendously benefit patient’s treatment outcomes and bring us one step closer to individualized medicine.

**Abstract:**

Despite the multiple preventive measures and treatment options, colorectal cancer holds a significant place in the world’s disease and mortality rates. The development of novel therapy is in critical need, and based on recent experimental data, cannabinoids could become excellent candidates. This review covered known experimental studies regarding the effects of cannabinoids on intestinal inflammation and colorectal cancer. In our opinion, because colorectal cancer is a heterogeneous disease with different genomic landscapes, the choice of cannabinoids for tumor prevention and treatment depends on the type of the disease, its etiology, driver mutations, and the expression levels of cannabinoid receptors. In this review, we describe the molecular changes of the endocannabinoid system in the pathologies of the large intestine, focusing on inflammation and cancer.

## 1. Introduction

According to the Canadian Cancer Society, cancer is the leading cause of death in Canada. More than 220,000 new cases were recorded in 2019, with lung, breast, colon, and prostate cancers as the most common [1]. Colorectal cancer (CRC) is the third leading cancer worldwide in men and women, with a 6% higher age-standardized incidence rate in men [2]. Each year, about one million individuals are newly diagnosed with CRC globally [3]. It is estimated that disease-related mortality in developed countries is around 33% [4]. Most sporadic cases occur in patients over 50, with 75% of cases occurring in those over 60. The lifetime risk of developing CRC is 3–4% in Western populations; however, if there is a family history of CRC, the risk is doubled [5].

In Europe and the USA, 17–71% of CRC is attributable to modifiable lifestyle factors [6]. The main environmental risk factors for CRC are smoking, obesity (with each unit of BMI increased, the risk for CRC increases 2–3%), type 2 diabetes mellitus, and alcohol consumption (20–50% increased risk) [6,7]. Aging is also an essential factor in CRC development [8]. In the study performed by Tomasetti et al. (2015), an analysis of 31 different tissues showed a positive correlation between the number of stem cell divisions and the lifetime risk for developing cancer [9]. Patients with inflammatory bowel diseases (IBDs), such as Crohn’s disease (CD) and ulcerative colitis (UC), are also at risk of developing CRC [10].

Additionally, in nearly 15–20% of CRC cases, there is a related hereditary factor. The most common hereditary-related CRC is Lynch syndrome, characterized by microsatellite instability (MSI) caused by mutations in mismatch repair genes, such as MLH1, MSH2, MSH6, and PMS2 [5]. The familial adenomatous polyposis that develops due to the APC gene mutation is the second most frequent familial CRC [11]. Despite the multiple preventive measures, screening procedures, and a wide variety of treatment options, CRC holds one of the most significant positions in world disease and mortality rates. The development of novel, more effective preventive measures and treatment approaches are critically needed, and based on recent experimental data, cannabinoids could potentially become good candidates [12,13,14,15,16,17,18,19,20].

For many centuries, cannabis plants have been used empirically to treat different diseases, including cancer [21]. Cannabinoids are commonly used as a treatment for insomnia, an appetite stimulator, or as an antinociceptive agent to alleviate chemo- and radiotherapy-induced nausea [22]. Since cannabinoids regulate CB1 expression, reduce the motility of GI tract, and decrease proinflammatory mediators, they could potentially become one of the treatments for IBDs [20,23,24,25]. Presently, there is broad scientific support regarding cannabinoid cytotoxicity in intestinal malignancies [12,16,26,27,28]. Some experiments indicated a higher potency of whole-plant extracts rich in phytocannabinoids, such as cannabidiol (CBD) and over purified cannabinoids [29]. Cannabinoids may reduce colonic polyp formation, intestinal inflammation, and reduce cancer cells growth via cannabinoid 1 (CB1), cannabinoid 2 (CB2) receptors, transient receptor potential cation channel subfamily V member 1 (TRPV1) receptors, and G-coupled protein receptor 55 (GPR55) [12,20,28,30,31,32,33,34,35,36]. The mechanisms of anticancer effects of cannabinoids include the activation of apoptosis, endoplasmic reticulum (ER) stress response, downregulation of survivin (inhibitor of apoptosis), and decrease of RAS/MAPK and PI3K/AKT signaling [12,16,26,28,31]. Despite the multiple possibilities of clinical applications for cannabinoids, there are few data regarding the anticancer effects of cannabinoid compounds in case reports [37,38,39]. In this review, we will focus on cannabinoid system regulation of normal and inflamed intestines, the anti-CRC properties of cannabinoids, and their place in CRC pathogenesis, prevention, and treatment.

## 2. Endocannabinoid System and Intestinal Homeostasis

Currently, there are three main known classes of cannabinoids: endocannabinoids that are present in the human body, phytocannabinoids that are extracted from the cannabis plant, and synthetic cannabinoids [21]. In this section we will discuss cannabinoids and ECS overall, their maintenance of intestinal homeostasis, as well as interactions with intestinal microbiota.

### 2.1. Introduction to the Cannabinoid System

To accomplish their action, cannabinoids mainly bind to seven-transmembrane G_i/o_-coupled receptors (GPCRs), usually the inhibitory type [21,40,41]. When cannabinoids bind to CB1 or CB2 receptors, there is a decrease in levels of cyclic adenosine monophosphate (cAMP) and expression of adenylate cyclase [40,41]. CB1 and CB2 receptors share only 44% of the protein homology and 68% in the transmembrane domains with binding sites for cannabinoids [42].

In in situ receptor/G protein reconstruction techniques, the activation of CB1 receptors results in high-affinity interactions with both G_i_ and G_o_, whereas CB2 activation results in high-affinity interaction with G_o_ [43]. For CB1 receptors, the synthetic compounds HU210, WIN 55,212-2, and endocannabinoid anandamide (AEA) have a maximum activation effect on the G_i_ cascade. The stimulation of G_o_ is achieved only by HU210, whereas AEA, Δ9-THC, WIN 55,212-2 only have a 60–75% effect on the G_o_ compared to HU210 (partial agonists) [43]. Similar data were obtained for CB2 receptors; in the ligand interaction experiments, CB2 stimulation is induced by HU210, which demonstrates maximal activation (a full agonist). Other compounds showed submaximal effects on CB2 receptors, with WIN 55,212-2 having 64%, AEA 42%, and Δ9-THC 44% (partial agonists) [43]. Such agonists induce different conformational changes in CB receptors, thereby allowing us to choose the CB targeted therapy that modifies G-protein signaling more selectively [43]. One of the pathway for cannabinoid action is comprised of GPCR kinase-3 and *β*-arrestin-2, which causes desensitization and relates to the development of tolerance that results in CB1 internalization [44]. Receptor desensitization is one of the molecular mechanisms that underlies the onset of cell tolerance to a particular stimuli [44] and can become one of the reasons for cannabinoid treatment resistance.

Ionic channels are placed in the membrane as multimeric complexes that form passage pathways for selected molecules triggered by mechanical or chemical signals. Cannabinoids can stimulate A-type potassium channels, which causes an increased efflux of potassium from the cell. Moreover, by binding to CB1 receptors, cannabinoids may inhibit N- and P/Q-types of voltage-dependent calcium channels and activate inwardly, rectifying potassium channels, which results in decreased calcium influx and increased potassium efflux from the cells [44,45,46,47,48]. The ionic channels that respond to cannabinoids are transient receptor ion channels (TRPs), such as TRPV1, TRPV2, TRPM8, and TRPA1 [45,46,47,48,49].

In addition to CB1 and CB2, there are multiple other receptors that may respond to cannabinoids [40]. The most studied receptors are GPR119, GPR55, peroxisome proliferating activated receptor *α* (PPAR*α*), and PPAR*γ* [35,49,50]. We will discuss some receptors and pumps listed in different sections throughout this review regarding their effects on CRC and intestinal inflammation.

### 2.2. Endocannabinoid System

The endocannabinoid system (ECS) includes CB receptors, cannabinoid enzymes, and endocannabinoids. Endocannabinoids are mostly represented by arachidonoyl ethanolamine, or anandamide (AEA), and 2-arachidonoylglycerol (2-AG), which are derivatives of membrane phospholipids [21,40] and are produced by multiple pathways. Thus, the inhibition of enzymes that take part in their synthesis will not always result in changes in endocannabinoid levels and may affect the amounts of other cell mediators [51,52]. Endocannabinoids are synthesized from cell membrane’s phospholipids on demand due to intracellular calcium elevation. Both endocannabinoids, AEA and 2-AG, signal through GPCRs, ion channels (TRPs), and nuclear receptors (PPARs) [53]. PPARs are the subfamily receptors that act with retinoic X receptors of the nuclear hormone receptor superfamily, regulating the expression of target genes by binding to peroxisome proliferator response elements in the genes [54].

The first endocannabinoid discovered was AEA [55]. AEA is a part of the larger family of N-acylethanolamines, and 2-AG belongs to a family of 2-acylglycerols. There are four main routes of AEA synthesis: *N*-acyl-phosphatidiyl-ethanolamine-hydrolyzing phosphatase D (NAPE-PLD) [56], NAPE-phospholipase C followed by phosphatase [57], dual hydrolysis of the acyl groups by the phospholipase D, and (Lyso)-*N*-acylphosphatidylethanolamine lipase (ABHD4) followed by hydrolysis by glycerophosphodiester phosphodiesterase 1 (GDE1) [58,59,60]. Alternatively, AEA can be produced by *α*/*β* lysosomal hydrolase 4. After AEA accomplishes its action, the cell reuptakes it for enzymatic degradation by fatty acid amid hydrolase (FAAH) [61], or monoacylglycerol lipase (MAGL) [62,63,64]. The third route of AEA degradation is COX-2, which results in the production of prostamides [65]. Finally, the fourth degradation process of AEA is via N-acylethanolamine-hydrolyzing acid amidase (NAAA) [66].

AEA is a partial agonist of CB1 receptors [51]. Furthermore, AEA binds to CB2 with low efficacy and can act as an antagonist of CB2. AEA is present in the extracellular space and is accumulated in cells via facilitated diffusion due to its transmembrane concentration gradient and does not require ATP and sodium [51]. In a low CB receptor expression, AEA can be an antagonist to high efficacy agents. However, CB2 receptors can be induced up to 100 fold by AEA, especially during tissue injuries or inflammation [67,68]. When CB1 receptors are expressed at a normal level, AEA can directly stimulate hydrolysis of PIP2, releasing inositol-1,4,5-phosphate (IP3), which eventually causes Ca^2+^ release from ER [69]. Activation of the PI3K pathway may indirectly affect levels of PIP2 and regulate calcium release within cells [70]. Except for CB1 and CB2 receptor interaction, AEA also binds to L-type calcium channels that can produce non-CB-mediated effects in vivo [71,72].

Another endocannabinoid, 2-AG, is produced from phospholipids in a two-step process. First, by the enzyme phospholipase C, 1,2-diacylglycerol is produced; next, diacylglycerol lipase (DAGL) converts it to 2-AG. The degradation of 2-AG is similar to AEA and it is held by MAGL, COX-2, lipoxygenase, or cytochrome P450 enzymes [73]. Additionally, 2-AG is an agonist of CB1 and CB2 receptors [68]. The experimental inactivation of endocannabinoid degradation pathways, such as blocking MAGL, may result in CB receptor desensitization in the tested tissues with subsequent elevation of 2-AG [74,75].

Knowledge of endocannabinoid’s synthesis and degradation process would help us understand the adaptational and pathophysiological changes of ECS in intestinal inflammation and malignancies, as well as develop newer and better treatment options for these diseases.

### 2.3. Normal Intestinal Cells and Their Functions

The normal large intestinal lumen consists of epithelial cells organized into anatomical structures called crypts of Lieberkühn [76]. Crypts are the source of intensively renewing and proliferating cells. The crypt base is composed of actively dividing stem cells, from which most intestinal cell lineages differentiate [4,76]. Colonocytes are the most common crypts cell type taking part in intestinal absorption. The other cells present in the crypts include mucin-producing Goblet cells and Tuft cells, which sense the intestinal content, enteroendocrine cells that produce hormones as a reaction to internal and environmental stimuli, and the microfold cells transporting luminal antigens [76,77].

The signaling pathway that plays a crucial role in the proliferation, maintenance, and differentiation of intestinal cells is Wingless-related integration site (WNT)/*β*-catenin. This pathway stimulates the division and differentiation of stem cells [4]. Moreover, it is important to emphasize that WNT, Notch, bone morphogenic protein (BMP), and transforming growth factor *β* (TGF-*β*) signaling are essential for the homeostasis of normal colon epithelial cells, and they are often dysregulated in intestinal malignancies [78]. Some studies have shown that aging is associated with increased genomic instability within intestinal crypts, even in the histologically healthy epithelium. Over time, deletions, translocations, duplications, and gene-conversions accumulate, forming a strong niche for CRC development [8,79].

### 2.4. Cannabinoids in the Gastrointestinal Tract

The ECS takes part in neuronal proliferation, differentiation, axon guidance, and synaptogenesis in many organs in the embryonic and early postnatal periods, including large intestines [80]. ECS regulate myenteric neural activity, the vagus nerve, sympathetic nervous system functions, and the release of ghrelin and cholecystokinin-8 in the gastrointestinal tract (GIT) [81]. Brain stem endocannabinoids modulate vagal control of GIT motility and emesis via CB1, CB2, and TRPV1 receptors [82]. The endocannabinoid expression levels in the gut and brain differ in states of satiety, diarrhea, emesis, and intestinal inflammation [82], pointing out the critical role of the ECS in the regulation of GIT homeostasis via the gut–brain axis and adaptation to pathological alterations in the local intestinal microenvironment.

#### 2.4.1. Endocannabinoid System in Intact GIT

GIT maintains the endogenous regulation of the ECS according to its needs [83]. As we previously mentioned, AEA and 2-AG are synthesized on demand from membrane lipids by intracellular calcium influx [84]. ECS maintains epithelial integrity, interacts with gut microbiota [85], and suppresses chronic stress-induced visceral hyperalgesia [82]. Typically, 2-AG and palmitoylethanolamide (PEA)—another endocannabinoid—are the “gatekeepers” of the intestines. Their actions are achieved by increasing intestinal barrier functions. On the other hand, AEA can work as a “gate opener” [85] that increases intestinal permeability. Throughout this review, we will discuss the role of the ECS regarding changes in epithelial barrier function in different pathological conditions, such as intestinal inflammation and cancer.

#### 2.4.2. Cannabinoid Receptors Expression in Intact GIT

Both types of CB receptors are present within the gut, but they are variously expressed and distributed in epithelial cells, lamina propria, smooth muscle cells, and enteric nervous plexuses. In the GIT, both CB1 and CB2 receptors are highly expressed in the enteric nervous plexuses, especially myenteric and submucosal ones [86]. The CB1 receptors are mainly present in excitatory motor neurons, interneurons, and intrinsic primary afferent neurons [87,88]. Within the intestinal lumen, CB1 expression is high on the crypts’ epithelial cells, goblet cells, and absorptive cells of the apical surfaces [89]. In contrast, CB2 receptors are absent in most normal intestinal epithelial cells, except for Paneth cells [90]. It was established that the highest levels of CB2 receptor expression in GIT have subepithelial immune cells, such as macrophages and plasma cells, which infiltrate intestinal lumen [89,91,92]. More to that, the expression of cannabinoid receptors and other components of ECS varies in the intact and diseased tissues [89].

In addition to CB1 and CB2 receptors, there are other types of receptors and pumps present throughout the gut, modulated by the ECS. For instance, endocannabinoid-like compounds PEA and oleoylethanolamide (OEA) act mostly on the GPR55 and GPR119 receptors present in the gut and can affect AEA signaling [93]. What is more, THC, AEA, OEA, and PEA activate transcription factor PPARα, resulting in a feeling of satiety and inhibition of inflammation [94]. PPARα is mainly present in nervous plexuses of GIT as well as in enterocytes [95].

Endocannabinoids (AEA, acylethanolamide, and OEA) and phytocannabinoids (CBD, CBG, THCV) can activate TRPV1 receptors, normally responding to high temperatures and protons [96,97,98,99]. TRPV1-4 are thermosensitive receptors, TRPV5 and 6 are non-thermosensitive receptors that allow the passage of calcium ions. They are activated by heat or decreased cell pH. TRPV1 and TRPV2 channels are present in dorsal root ganglia cells of the gut. The highest density of TRVP1 is in the myenteric plexus and inter-ganglionic fibers [100]. TRPV2 is expressed in immune cells, such as neutrophils, macrophages, and monocytes. These ionic channels help regulate intestinal motility, heat-induced cellular effects, and stimulate migration and phagocytosis by the immune cells. In the gut, TRPV3 is widely distributed, especially in the epithelial cells of the distal colon, ileum, and jejunum [101]. Activation of TRPV2 in non-malignant cells induces their translocation to the plasma membrane, which stops cell proliferation and induces cell death [102,103]. Overall, these channels mediate the flux of cations (calcium, sodium) down their electrochemical gradients [101].

#### 2.4.3. The Microbiome and the Cannabinoid System

The intestinal microbiota plays a crucial role in intestinal homeostasis. The endocannabinoids maintain GIT homeostasis by regulating appetite, metabolism, and interaction with intestinal microorganisms via cannabinoid receptors [85,104,105]. Changes in the microbiome can lead to increased intestinal wall permeability and inflammation [106]. In this section, we will discuss how gut microbiota interacts with ECS and how these interactions help maintain intestinal homeostasis.

Human GIT is dominated by the phyla *Bacteroides*, *Firmicutes*, *Proteobacteria*, and *Actinobacteria*. There are many lactic acid fermenting bacteria, such as *Lactobacillus* and *Bifidobacterium*. Most bacteria play a beneficial role in our GIT, but some can induce pathogenic processes that can later promote colon carcinogenesis [107]. Studies show that 10% of the human transcriptome is regulated by microbiota [108]. Some bacteria, such as *Akkermansia muciniphila* and *Bifidobacterium*, mediate multiple interactions between the gut microorganisms [109]. Changes in the levels of gut microbiota or in their diversity can eventually lead to metabolic disorders and intestinal inflammation [110,111].

The microbiota’s ability to regulate the production of cytokines and activate transcription factors can contribute to intestinal tumorigenesis; microbiota can also play a vital role in colitis-associated cancer (CAC) development [112]. To modulate intestinal homeostasis, microbiota acts through damage-associated molecular patterns (DAMPs), toxins, metabolites that act on toll-like receptors (TLRs), and nucleotide oligomerization domain-like receptors that are present on colonocytes and immune cells. Binding to TLR4 can stimulate the NFκB transcription factor and subsequent production of pro-inflammatory cytokines, such as IL-6, TNF-*α*, and IL-1*β*. Intestinal metabolites produced by the interaction of microbiota with the gut, such as secondary bile acids, H2S, and amines, can induce DNA damage, contributing to the activation of inflammatory cascades with the production of phospholipase A2 and prostaglandin E2 (PGE2) [113]. Inflammation also increases intestinal permeability, causing a loss of E-cadherins between epithelial cells, allowing bacteria and toxins to enter the submucosa. Additionally, some bacteria activate STAT3 signaling, promoting the release of IL-17. Next, IL-17 stimulates T-helper 17, which produces IL-23. IL-23 maintains chronic inflammation and attracts neutrophils, which release reactive oxygen species (ROS) that result in a sustained chronic inflammatory response. Additionally, there is also a stimulation of the WNT/*β*-catenin pathway that activates colon cell proliferation and formation of intestinal adenomas and carcinomas [104,107].

##### Gate Keepers/Openers

Changes in gut microbiota and innate immune response could be linked to ECS [114]. For instance, the deletion of the myeloid differentiation’s primary response protein (MyD88) in high-fat diet mice, which takes part in Toll-like receptor (TLR) signaling, protected the mice from obesity, diabetes mellitus, and inflammation [114]. TLR stimulation leads to activation of IL1R-associated protein kinases and TNF-receptor-associated factor 6 that results in release of proinflammatory cytokines [115]. Thus, MyD88 deletion helped to maintain the intestinal barrier function, and elevated levels of regulatory T-cells in the intestinal wall [114]. These effects were accompanied by decreased levels of AEA and increased 2-AG, as well as higher expression of GPR119 in the intestines of the mice fed a high-fat diet [114]. By deleting the MyD88 in intestinal epithelial cells, the host’s microbiome underwent changes that resulted in elevated levels of gatekeeping endocannabinoids, such as 2-AG [114]. Additionally, the deletion of MyD88 in intestinal epithelial cells causes increased endocannabinoids that result in anti-inflammatory effects (reduced levels of IL-6 and resistin) in intestines and increased levels of regulatory T-cells in high-fat diet obese mice [114]. This study further supports the protective role of some endocannabinoids against the increase of intestinal permeability.

One of the mechanisms by which microbiota can directly interact with the ECS is via the production of molecules like endocannabinoids, which bind with the same receptors as cannabinoids and promote metabolic disorders [106]. Bioinformatics analysis of human microbiota showed that commensal intestinal bacteria, which produce high levels of N-acyl amides, interact with lipid-like GPCRs that regulate GIT physiological processes. Cell-based and mice models showed that bacteria which produce GPR119 agonists can regulate metabolic hormones and blood glucose levels. This molecular mimicry of human ligands may open a new mechanism of regulation of host cellular responses via changes in the expression of bacterial genes (microbiome-biosynthetic gene therapy) [106].

Furthermore, gut bacteria can produce OEA and PEA in response to dysbiosis and decrease intestinal permeability via interaction with TRPV1 and PPAR*α* receptors [53,116]. These changes do not allow microbial toxins, such as LPS, to pass into the bloodstream, contributing to the prevention of metabolic disorders associated with obesity [117]. Moreover, microbiota can regulate the levels of expression of NAPE-PLD, CB1, FAAH, and AEA in obese mice, which results in increased adipogenesis [105]. The diet-induced obesity within in vivo models can be attenuated by the blockage of CB1 receptors, accompanied by decreased trafficking of macrophage type 1, and levels of inflammatory cytokines, such as IL-17, monocyte chemoattractant protein-1, eotaxin, and macrophage inflammatory protein-1*α* in adipose tissue [118]. As a result, the decreased inflammatory response is associated with lower intestinal permeability, decreased endotoxemia, and insulin resistance in treated mice [118].

In the case of GIT pathologies, the gut microbiota can undergo changes in their composition, causing endocannabinoids such as AEA to act as “gate openers”, resulting in increased intestinal permeability [119]. However, the pro-inflammatory effects of cannabinoids, such as AEA, could be reversed by the administration of bacteria *Akkermansia muciniphila* to the high-fat diet mice. This showed to reduce endotoxemia via increasing levels of gatekeeping endocannabinoids, such as 2-AG, 2-OG, and 2-PG [120]. In the high-fat diet model, there are amplifications of low-grade inflammation that eventually lead to dysbiosis and enhances CB1 expression, which has been shown to stimulate more metabolic disorders [53]. The blockade of CB1 receptors ameliorates dysmetabolism and dysbiosis in obese mice by increasing the amount of *Akkermansia muciniphila*, which has beneficial effects on metabolic rates and reduction of inflammation [118]. Blocking CB1 receptors is associated with an increased number of *Akkermansia muciniphila* and reduced *Lanchnospiraceae* and *Erisypelotrichaceae* in the GIT of experimental mice [118]. Moreover, continuous administration of THC causes CB1 receptor internalization and desensitization [121]. Thus, decreased CB1 expression can be associated with obesity, metabolism disorders leading to dysbiosis, and induced inflammation in the gut [122]. The addition of prebiotics, probiotics, and antibiotics into *ob/ob* mice models resulted in the modulation of CB1 receptors expression [119]. The blockade of CB1 can partially restore the distribution of zonula occludens 1 (ZO-1) and occludin in the intestines [105], which indicates the role of ECS in intestinal barrier function and may also suggest mechanisms for the epithelial–mesenchymal transition of CRC cells.

##### Modifiers of CB Expression

ECS’s protective role against GIT pathologies may be achieved via interaction with intestinal microbiota and subsequent modulation of the gut’s pro-inflammatory immune reactions. Commensal bacteria and pathogenic microbiota can reach mucosal and submucosal layers in intestinal walls, which can induce an inflammatory response. There are often upregulations of the CB2 receptors at the epithelial breakage sites, revealing their protective role against pro-inflammatory stimuli. Activation of CB receptors decreases leukocyte infiltration, inhibits cytokine release, and suppresses adhesion and migration of leukocytes to damaged sites [123]. Moreover, activation of CB2 receptors in afferent nerves of the GIT tends to reduce visceral pain and intestinal motility. Additionally, with activation of the central nervous system, CB1 receptors decrease emesis [124]. Scientists demonstrated the important role of probiotics in intestinal homeostasis [125]. The addition of the probiotic *Lactobacillus acidophilus* has been associated with increased expression of enterocytic CB2 receptors in treated animals [92,126]. The oral administration of *Lactobacillus acidophilus* to rats with experimental IBD induced the expression of μ-opioid and CB2 receptors that mediated the analgesic effect, which is like the action of morphine [126].

Intestinal microbiota regulates ECS tone in the gut, which results in changes in intestinal permeability and plasma lipopolysaccharide (LPS) levels [105]. The GIT microbiome can modulate CB1 receptor expression in normal and obese mice. In obese mice fed with prebiotics, the AEA expression levels were decreased, and FAAH expression was elevated. In contrast, 2-AG did not change despite the decrease in MAGL mRNA expression. In obese mice, the LPS levels were reduced by the prebiotic treatment, which correlated with levels of CB1 and AEA expression in the colon [105]. These data support the hypothesis of protective effects of ECS in inflammation and obesity, which can be regulated by the microbiota.

Ingestion of cannabinoids, especially CB1 agonists, may induce appetite; however, obesity is lower in chronic cannabis users. Chronic administration of THC can reduce weight gain and energy intake in diet-induced obese mice, but not in lean mice. It was also shown that changes in gut microbiota contribute to the effects of THC on adipogenesis. In a high-fat diet, the ratio of *Firmicutes* to *Bacteroides* increased, which can be prevented by chronic THC administration and eventually may decrease food intake and weight gain [121]. Gut microbiota can decrease CB1 expression in adipocytes, which controls adipogenesis. In the adipose tissue of experimental mice, there were increased expressions of *N*-acylphosphatidylethanolamine-preferring phospholipase D (NAPE-PLD) and reduced levels of FAAH expression, which led to significant elevation of AEA levels. Treatments using prebiotics reduced AEA levels in mice adipocytes by activating one of the endocannabinoid’s degrading enzymes, FAAH. Moreover, administration of *Lactobacillus acidophilus* increased CB2 receptors expression [105]. These results showed us how important it is to keep a stable relationships between the gut microbiota and the host’s immune system—to maintain beneficial symbiotic relationships between the host and microbial community to prevent inflammation, metabolic disorders, and obesity [118].

Overall, the variability of intestinal microflora is necessary to maintain normal homeostasis of the intestines [127]. The presented data show that ECS reacts to intestinal damage, decreases inflammatory reactions, and can interact with GIT microflora. Thus, ECS has mainly a protective effect on gut mucosa.

## 3. Cannabinoids in Intestinal Inflammation

During colonic inflammation, enterocytes can produce mediators that potentiate the disease’s progression and may enhance intestinal damage [83]. The importance of persistent chronic inflammation in the GIT lies in its possible transformation into CRC. The clinical data show that patients with IBD have a two-fold higher risk of CRC development than the general population. It is estimated that the mutations associated with UC are in the TP53, KRAS, and SMAD4 genes and are commonly observed in colitis-associated CRCs (CACs) [128,129,130]. The same group of mutations were found in the nonaffected surrounding epithelium of UC-associated cancers from a process called “field cancerization”. This phenomenon can explain the appearance of synchronous and metachronous tumors present in IBDs [131]. Consequently, it is crucial to understand the molecular pathways involved in chronic inflammatory pathologies of the GIT to treat the damage caused by inflammation and prevent the risk of CAC development.

Since the ECS is critically important in response to intestinal inflammation and its regulation, a thorough examination of its alterations could reveal new treatment options for gut inflammatory diseases. ECS plays an essential role in reducing intestinal immune reactions, including innate and adaptive immune responses by various mechanisms of action (see Table 1) [132].

### 3.1. Colitis and Changes in ECS

Intestinal biopsies of patients with different inflammatory diseases such as IBDs, diverticulitis, and celiac disease revealed higher expressions of cannabinoid receptors and endocannabinoids than in intact intestinal tissues. The experiments provided by D’Argelio et al. (2006) showed that there is a two-fold elevation of AEA in patients with untreated UC, which can correlate with the activity of the disease [133]. Transcriptome analysis performed by Grill et al. (2019) revealed that AEA, OEA, and 2-AG were elevated in patients with IBDs; however, only PEA and OEA were elevated in CRCs [20]. Additionally, in the CD study group, CB1 and GPR119 receptor transcription were significantly lower, emphasizing the protective role of cannabinoid receptors in intestinal inflammation [20]. In contrast, the CB2 expression in CD was increased, especially in the damaged intestinal crypts [91,134]. Other experiments showed higher levels of CB2, DAGL*α*, and MAGL expression in mild and moderate forms of colitis, although in the light type of intestinal inflammation, CB1, CB2, and DAGL-*α* were decreased and NAPE-PLD was elevated, especially in patients treated with aspirin or aspirin and corticosteroids. At the cellular level, MAGL and FAAH were particularly increased in the immune cells during acute pancolitis, but dropped after treatment with non-steroidal anti-inflammatory drugs (NSAIDs) [89]. Thus, studies showed that in case of light forms of colitis there were increased expressions of CB receptors and higher productions of endocannabinoids; however, in moderate types of colonic inflammation caused by IBDs, CB1/CB2 expression decreases, as do endocannabinoid levels.

Some studies pointed out that the crucial response of GIT to ECS activation depends on CB1 receptor expression levels. These receptors mediate protective reactions against colonic inflammation. In animal models representing colitis-associated tumors, pharmacological stimulation of CB1 and CB2 receptors alleviated signs of experimental bowel inflammation [133,135,136]. For instance, in the mustard oil colitis model, direct activations of CB1 and CB2 receptors by their agonists (arachidonoyl-chloro-ethanolamide and JWH-133) revealed their protective roles on intestinal mucosa [137]. The inhibition of cannabinoids degrading FAAH and MAGL enzymes was shown to be protective against colitis induced by dextran sulfate sodium (DSS) and trinitrobenzene sulfonic acid (TNBS) [138]. Intrarectal infusion of 2,4-dinitrobenzene sulfonic acid (DNBS) and oral administration of DSS, which promotes the development of colitis in animals [135], showed that in CB1-deficient mice, there was a significantly stronger inflammatory response to experimental colitis than there was in the wild type [135]. Moreover, treatment of wild-type mice with cannabinoid antagonist SR141716A mimicked the CB1-deficient phenotype, and administration of CB1 receptor agonist HU210, or genetic inhibition of FAAH, protected against DNBS-induced colitis in the mice [135]. The synthetic CB receptor agonists WIN 55,212-2 had protective roles in DSS-induced colitis by inhibiting the p38/MAPK pathway [139], which may have significantly suppressed pro-inflammatory responses in the GIT. The experiments on intestinal epithelial cells, described by Izzo et al. (2009), have shown that cannabinoids also induced wound healing by CB1 activation and suppression of cytokine release via CB2 receptors [134]. Moreover, in the CB1-deficient mice, the electrophysiological recordings of circular smooth muscles showed spontaneous oscillatory action potentials, indicating the role of CB1 in early induced irritation of the intestines [135], and release of acetylcholine in the myenteric plexus that innervates the gut, leading to decreased intestinal motility [140]. In croton oil-induced ileitis, CB1 expression was high, which is associated with reduced transit time [124]. In contrast, LPS-induced intestinal propulsions were ameliorated by the induction of CB2 receptors [141]. Thus, activation of CB1 and CB2 receptors decreased visceral sensitivity due to colonic distention and inflammatory stimuli [142], and could reduce intestinal contractility [134,140,143].

### 3.2. Gatekeeping Mechanisms of ECS

The previously mentioned “gatekeeping” mechanism of endocannabinoids in the intestines is achieved by suppressing cell-mediated immunity through T-helper 1 cells and stimulating humoral immune response via T-helper 2 cells. These effects are mostly regulated by CB2 receptors signaling [132,144]. Animals with deficient CB2 receptors had decreased levels of intestinal natural killer cells and CD4+ memory cells [145], which may favor the development of intestinal malignancy due to the evasion of adaptive immune response [146]. The immunomodulatory role of cannabinoids was shown in experiments with the administration of Δ-9-THC into mice followed by infection with *Legionella pneumophila*, which resulted in inhibition of T-helper 1 activation. The effects of Δ-9-THC were accompanied by the suppression of cytokines such as IL-12 and IFN-*γ*, and increased levels of IL-4. Such changes of immune signaling molecules stimulated humoral immune response and suppressed cellular immune response. Additionally, CB1 agonist SR 141716A attenuated inhibition of IL-12*β*, and CB2 agonist SR144528 increased GATA3 mRNA expressions in the mice’s spleen. These results indicated an enhancement of T-helper 2 cells upon cannabinoid treatment [132]. In another study, the plant-derived THC had an inhibitory effect on TNF-*α*-induced release of pro-inflammatory cytokine IL-8 in HT-29 CRC cell line [147]. In addition, endocannabinoids played a pertinent role in maintaining immune tolerability by controlling the expansion of a regulatory pool of T lymphocytes and suppressive CX3CR1^hi^ macrophages [85,148]. Macrophages and plasma cells in the human large intestine express both CB1 and CB2 receptors that take part in immune reactions [91]. In the inflamed gut, activated macrophages, monocytes, and dendritic cells increased the production of endocannabinoids (AEA, 2-AG) and may reduce increased gut permeability caused by pro-inflammatory stimuli [149].

Other in vitro experiments supporting the hypothesis of the barrier-maintaining function of ECS in the gut showed that OEA could modulate permeability of intestinal cells. PEA is a ligand to PPAR-*α* with anti-inflammatory and analgesic action [54]. PPAR-*α* reduces inflammation via induction of IkB-*α* that inhibits nuclear translocation of NFκB, which is a pro-inflammatory transcription factor that induces TNF-*α* expression, which recruits immune cells to the damaged tissues [150]. PEA is an endocannabinoid-like compound synthesized from membrane phospholipids via N-acyltransferase and NAPE-PLD. The degradation pathway of PEA is maintained by FAAH and N-acylethanolamine-hydrolyzing acid amidase (NAAA) [151]. NAAA is highly expressed in intestinal leukocytes [152], and its inhibition can alleviate intestinal inflammation [153].

**Figure 1:**
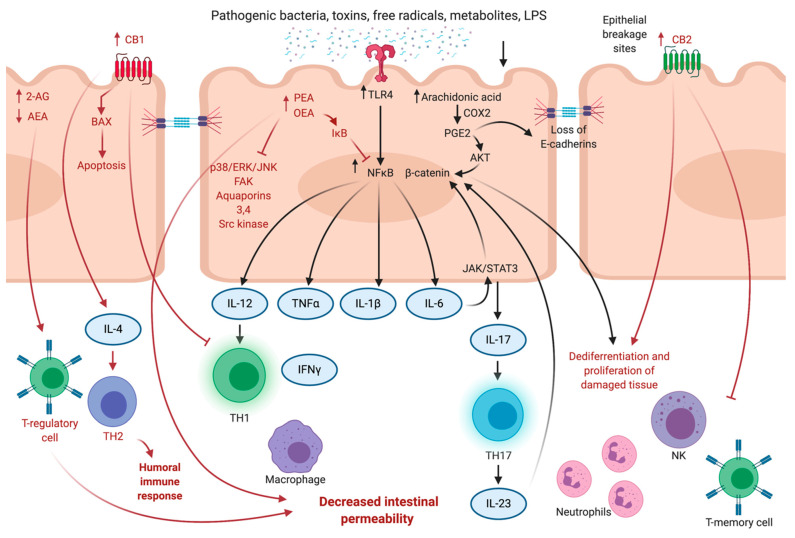
The protective effect of endocannabinoid system in intestinal inflammation. See text for discussion. Created with BioRender.com (accessed on 10 August 2021). Abbreviations: 2-AG—2-arachidonoyl glycerol; AEA—anandamide; CB1—cannabinoid receptor 1; CB2—cannabinoid receptor 2; COX2—cyclooxygenase 2; IFN-*γ*—interferon *γ*; ERK—extracellular regulated kinase; FAK—focal adhesion kinase; IL-1*β*—interleukin 1*β*; IL-6—interleukin 6; IL-10—interleukin 10; IL-12—interleukin 12; IL-23—interleukin 23; NFκB—nuclear factor κB; STAT3—signal transducer and activator of transcription 3; PGE2—prostaglandin E2; PLC—phospholipase C; TH1—T-helper 1; TH2—T-helper 2; TH17—T-helper 17; TLR 4—Toll-like receptor 4; TNF-*α*—tumor necrosis factor *α*.

One of the underlying mechanisms of how cannabinoids achieve anti-inflammatory and anti-tumor effects in the large intestine is by alleviating the inflammatory-related release of TNF-*α*, IFN-*γ*, IL-1*β*, and IL-6 mediators, which shows their protective role against intestinal inflammatory diseases and CRCs [83] (see Figure 1). The large intestines’ inflammatory processes are associated with glial cell responses by the expression of Toll-like 4 receptors (TLR4) and S100B [151,154]. In DSS-induced colitis, the anti-inflammatory effect of PEA is mediated via inhibition of enteroglial-specific S100 protein. The S100 protein promotes macrophage recruitment in the intestinal mucosa, induces an inflammatory response, and interacts with TLR4. PEA targets the S100B/TLR4 axis through PPAR-*α*, which results in inhibition of NFκB in enteric glial cells through p38/ERK/JNK signaling [154]. Endocannabinoids PEA and OEA act as agonists of GPR55 (PEA) and GPR119 (OEA) to enhance the immune effects of AEA [155,156]. In DSS and DNBS models of IBDs, PEA administration reduced microscopic signs of inflammation, neutrophil infiltration, and COX-2, PGE2, and inducible nitric oxide synthase (iNOS) expression via CB2, GPR55, PPAR-*α* signaling, and TRPV1 channels [157]. Thus, the addition of PEA attenuated intestinal permeability and inflammation as well as increased colonic cell proliferation by elevating TRPV1 and CB1 expression levels [157]. The antiproliferative effects of PEA on cancer cells was achieved by elevation of AEA levels within the cells due to inhibition of FAAH. Subsequently, the elevated levels of AEA caused stimulation of vanilloid receptors type 1 [158,159]. It was also shown that OEA and PEA increased transepithelial electrical resistance of Caco-2 cells by 20–30% via TRPV1 and PPAR*α* receptors. OEA and PEA may induce cytoskeletal changes and activate focal adhesion kinase (FAK) and ERK1/2 and as a result, decrease epithelial permeability. These endocannabinoid-like compounds also reduce levels of Src kinases, aquaporins 3 and 4, and activated potassium channels [116]. Downregulation of main degrading enzyme FAAH results in the elevation of OEA and PEA, leading to a significant decrease in intercellular permeability. OEA acts via TRPV1 (apical application) and PEA (basolateral membrane application) through PPAR-*α* receptor signaling [116]. Furthermore, in experiments provided by Alhouayek et al. (2015), endocannabinoid PEA inhibited inflammation-associated angiogenesis via CB1, CB2, GPR55, PPAR-*α*, and TRVP1 receptor interactions [153].

GIT plays an essential role in systemic inflammatory response, endotoxemia, and sepsis due to its barrier protective function against translocation of bacteria and their toxins into the bloodstream [115]. Thus, maintaining gatekeeping property of the intestines in some cases can help preventing the development of toxin-induced multiorgan failure [115]. The study performed by Espinosa-Riquer et al. (2019) proposed that 2-AG, together with CB2 receptors, play a role in the development of endotoxin tolerance in mice bone-marrow-derived mast cells [160]. Mast cells can become hyporeactive if TLR4 receptors are stimulated by endotoxins such as LPS during prolonged periods of time. The authors showed that 2-AG administration induced endotoxin tolerance via inhibition of NFκB pathway and TNF secretion. Additionally, the production of 2-AG is TLR4 dependent, which further indicates that 2-AG participates in negative autocrine regulation of mast cells activation. More to that, inhibition of CB2 receptors prevented development of endotoxin tolerance. Thus, mast cell-related branch of innate immune response can be partially regulated by ECS [160]. Another study showed that CB1 and CB2 agonism prevented LPS-induced GIT motility and reduced IL-6 levels [161]. In mice model of LPS-induced sepsis, addition of CBD caused decrease of S100B protein levels, which consequently abrogated the hyperactivation of intestinal glial cells, macrophages, and mast cells [162].

On the other hand, elevation of levels of endocannabinoids such as AEA and 2-AG can lead to concentration-dependent increase of intestinal permeability in inflammation and hypoxia via CB1 receptors activation [163]. In Caco-2 cells, 2-AG levels elevated under hypoxic conditions and inflammatory responses. In human intestinal samples, there are increased macrophage colony-stimulating factor IL-12, IL-13, and IL-15 under normal, inflammatory, and hypoxic conditions [163]. The study by Karwad et al. (2017) showed that AEA and 2-AG played an important role in intestinal permeability in vitro and ex vivo [163]. However, the intraperitoneal administration of AEA in mice with TNBS-induced colitis decreased macro- and microscopic scores of colitis and reduced immune cell infiltration. Additionally, inhibition of FAAH decreased the accumulation of inflammatory cytokines and inhibited leukocyte proliferation in DNBS-experimental colitis [164,165]. Another experiment showed the pro-inflammatory effect of AEA via TRPV1 receptors. AEA has been proven to elevate myeloperoxidase activity in the rat ileum. Thus, it can become pro-inflammatory; however, to activate TRPV1 pumps, AEA concentrations should be much higher than those needed for CB receptors activation [96].

### 3.3. Anti-Inflammatory Effects of Phytocannabinoids

One of the studies showed that ingestion of high-tetrahydrocannabinolic acid (THCA) cannabis extract caused inhibition of COX-2, matrix metalloproteinase-9 (MMP-9), and alleviated inflammatory response via GPR55 receptors, resulting in anti-inflammatory effects in cell culture experiments and in IBD patients’ biopsy material [34]. The study from Nallathambi et al. (2017) showed significant anti-inflammatory effects of high-THCA cannabis extract achieved via GPR55 receptor signaling, leading to a decrease of IL-8 expression in TNF-*α*-pretreated HCT-116 CRC cells [34]. Addition of GPR55 antagonists reduced the anti-inflammatory activity of the high-THCA fraction of *Cannabis sativa* extract [34]. In DNBS experimental colitis, another phytocannabinoid, cannabigerol (CBG), can inhibit cytokines and ROS production as well as suppress macrophage and mast cell migration by binding to CB2 receptors [166]. Another phytocannabinoid, CBD, was shown to reduce inflammation in LPS-induced colitis and UC patients via PPAR-*γ* receptors by inhibition of TNF-*α*, caspase 3, and iNOS [162]. Additionally, Jamontt et al. (2010) have demonstrated that THC and CBD also reduces signs of TNBS-induced colitis in rats [167]. The anti-inflammatory actions of CBD are exerted by the antagonistic effect on GPR55 [35]; activation of PPAR-*γ* and TRPV1 and simultaneous inhibition of FAAH result in elevated endocannabinoid levels [168]. A recent study showed that CBD within *Cannabis sativa* extracts has a higher anti-inflammatory effect on the gut than pure CBD [30].

Changes of immune response due to cannabinoid system activation may reduce intestinal inflammation and be beneficial in different types of colitis, including IBDs. However, in the case of CRCs, the shift of immune response from cellular to humoral can be one of the mechanisms whereby cancer cells evade immune responses, which contributes to their survival and cancer cell progression.

**Table 1:** The effect of cannabinoids in experimental models of inflammatory intestinal diseases.

Source of Study	Cannabinoid Receptors	Endocannabinoids	Changes in Endocannabinoid Synthesis	Changes in Endocannabinoid Degradation	Methods of Analysis	Effects	References
Human intestinal biopsies of CD	CB1, GPR55, GPR119 decreased; PPAR*δ*, TRPV1 increased	OEA elevated	DAGL-*α* increased	FAAH, NAAA increased	mRNA levels	Correlates with disease severity	[20]
Human intestinal biopsies of UC	CB1, CB2, GPR119, PPAR*α*, PPAR*γ*, GPR18, GPR55 decreased; PPARδ, TRPV1 increased	AEA, OEA, and 2-AG elevated	NAPE-PLD decreased	FAAH decreased	mRNA levels	Correlates with disease severity	[20]
Human colonic biopsies of UC. Acute mild/moderate colitis	Increased CB2	-	DAGL*α* increased, NAPE-PLD decreased	FAAH, MAGL increased	Western blot and immunohistochemistry	CB2 signaling reduces colitis-associated inflammation	[89]
Human colonic biopsies of UC Quiescent pancolitis	CB1, CB2 decreased	-	DAGL*α* decreased, NAPE-PLD elevated	FAAH decreased	Western blot and immunohistochemistry	CB2 signaling reduces colitis-associated inflammation	[89]
DNBS, TNBS colitis, human UC biopsies	CB1/CB2 increased	AEA elevated	-	FAAH increased	Chromatography/mass spectrometry	Anti-inflammatory action	[133]
DNBS-induced colitis in mice	Increased CB1 expression, and CB1 stimulation	Treatment with CB1 agonist HU210	-	FAAH experimental genetic ablation	mRNA levels	Alleviates intestinal inflammation	[135]
DNBS-induced colitis in mice	TRPV1 and GPR55 downregulation	Increased PEA	NAPE-PLD not changed	NAAA, FAAH not changed	Immunohistochemistry, mRNA, liquid chromatography, and mass spectrometry	Decreased intestinal permeability	[157]
DNBS experimental colitis	CB2 stimulation	CBG treatment	-	-	mRNA levels	Anti-inflammatory effect	[166]
TNBS-induced colitis in mice	CB2 increased	Addition of CB2 agonists JWH133, AM1241	-	-	mRNA levels	Protects against inflammation	[136]
TNBS- and DSS-induced colitis in mice	Increased PPAR-*α*	AEA increased; PEA treatment	-	Inhibition of NAAA	HPLC-mass spectrometry, mRNA	Reduction of inflammation	[153]
Mustard oil and DSS-induced colitis in mice	CB2 increased expression (higher in mustard oil colitis than in DSS-induced colitis)	CB1, CB2 stimulation with arachidonoyl-chloro-ethanolamide and JWH-133	-	-	Immunohistochemistry (protein levels)	Alleviates intestinal inflammation	[137]
DSS and TNBS-induced colitis in. mice	-	-	-	FAAH inhibition	mRNA levels	Protective on colonic mucosa	[138]
DSS-induced colitis in mice	CB1 increased expression	Addition of CB receptor agonists WIN 55,212-2	-	-	Protein levels	Protective effect on colonic mucosa	[139]
TNBS-induced colitis, DSS-induced colitis	-	Addition of AEA	-	Inhibition of FAAH	Microarray analysis, miRNA expression, liquid chromatography/mass spectrometry	Decreased macro- and microscopic signs of colitis	[164,165]
TNBS-induced colitis in rats	Inhibition of GPR55, activation of PPAR-*γ*, TRPV1	THC, CBD	-	Inhibition of FAAH	-	Anti-inflammatory	[167]
Croton oil-induced ileitis in mice	CB1 increased expression	No significant change in AEA and 2-AG levels. Addition of CB receptor agonist CP 55,940 and CBN	-	-	HPLC, protein levels	Reduced intestinal motility	[124]
LPS-induced intestinal propulsions	CB2 induction	CB2 induction by JWH-133	-	-	-	Reduced transit time	[141]
LPS-induced colitis and intestinal biopsies from patients with UC	PPAR-*γ* activation	CBD treatment	-	-	-	Anti-inflammatory, decreased reactive gliosis	[162]
Caco-2 CRC cells	OEA acts on TRPV1 and PEA acts on PPAR-*α* receptor signaling	OEA and PEA treatment	-	Inhibition of FAAH	Liquid chromatography-mass spectrometry	Increased transepithelial electrical resistance and decreased intercellular permeability	[116]
Caco-2 CRC cells	CB1 activation	2-AG treatment	-	Inhibition of FAAH	Liquid chromatography-mass spectrometry	Increased intestinal permeability under inflammation and hypoxia	[163]
Human tissue biopsies of IBD patients, HCT-116, HT-29, and Caco-2 CRC cell lines	GPR55 stimulation	High-THCA cannabis extract	-	-	mRNA levels	Anti-inflammatory effect	[34]
Human intestinal biopsies from normal mucosa, intestinal adenomas, colorectal carcinomas, CRC cell lines	CB1 and CB2 stimulation	2-AG, AEA are 2- and 3-fold higher in adenomas and carcinomas	-	Increased FAAH in CRC	Liquid chromatography/mass spectrometry, mRNA levels, western blotting	Anti-cancer effect	[15]

## 4. CRC and Colonic Inflammation

CRC is not a single disease but rather a group of molecularly heterogeneous pathologies with standard features primarily affecting the colorectal region [4]. The “classic” steps in CRC “evolution” start from a polyp or aberrant cryptic foci (ACF) developing into early adenoma (<1 cm) followed by late adenoma (>1 cm), eventually transforming into adenocarcinoma, which takes around 10–15 years to develop [4]. The conventional adenoma-to-carcinoma model proposed by Fearon and Vogelstein (1990) was one of the first to explain the step-by-step processes of CRC development and is true for about 60–65% of colon cancers [169]. In this model, the primary initiation of the mutation is the downregulation of the APC gene, leading to an overgrowth of intestinal epithelial cells and the formation of colon adenomas. Further development in the model consists of mutations and epimutations in KRAS and NRAS, affecting mitogen activated protein kinase (MAPK) pathway; then SMAD4 or SMAD6 genes, affecting phosphoinositil-3-kinase (PI3K) pathway; and finally, downregulation of “guardian of the genome” p53, causing adenocarcinoma to progress [169]. Schepers et al. (2012) have been able to visualize and monitor a candidate stem cell for intestinal adenomas, which suggested that tumor cells from the intestinal crypt stem cell (Lgr5^+^) are the cells that power the growth of intestinal adenomas [170]. As some data show that the pro-inflammatory PGE2 stimulates tumor progression via stimulation of stem cells in the intestines. This can partially explain why NSAIDs have a protective role against colon carcinogenesis [171].

CAC develops in 20% of IBD patients, approximately after 30 years of disease onset [172]. Despite considerable similarities in the pathogenesis of CAC and “classical” CRC, there are still some differences. As previously mentioned, IBDs have a persistent chronic inflammatory response with increased levels of TNF, IL-17, IL-23, IFN-*γ*, and IL-6 due to activation of transcription factor NFκB and STAT3, which can lead to the formation of ACF and adenomas. Moreover, continuous activation of COX-2 increases KRAS signaling and promotes tumor survival, progression, and metastatic potential [173,174,175]. Increased levels of PGE2, which is produced from arachidonic acid, and protein kinase B (AKT) signaling increase intranuclear levels of transcription factor *β*-catenin that stimulate the proliferation of enterocytes [176,177]. Activating genetic alterations leading to nuclear accumulation of *β*-catenin (TCFZL2, FZD8, AX1N1) is seen in 41% of these neoplasms [129]. Additionally, in intestinal adenomas, there are higher TGF-*β* receptors expression and inactivation of p53 and BAX, leading to increased levels of COX-2 [178,179]. In many CRC tumors, there are infiltrations of natural killers, neutrophils, dendritic cells, and macrophages [180]. A vital difference between CAC and “classical” CRC is the infiltration in the intestinal wall, with T-cells that react with tumor-specific antigens in the classical type. However, in CAC, many T-cells are reactive against intestinal microflora. That is why, in CACs, CD8+ cells can stimulate tumor proliferation with their cytokines [175], and in this case, the immuno-suppressive effect of cannabinoids may have a protective role against cancer development and progression.

Another study associated with exploring the differences in the genetic landscape of CACs and classical CRCs showed that predominant mutations are in genes responsible for cell motility and cytoskeletal proteins such as RAC1, DOCK2, DOCK3, PREX2, and RADIL. In some cases, chromatin modifiers and epigenetic regulators EP300 and TRRAP are also preferentially changed [181]. Additionally, IBD-associated cancers have a lower frequency of APC mutations [129]. Furthermore, one of the studies performed by Schwiebs et al. (2019) revealed the role of sphingolipid deterioration in CAC. Sphingosine-1-phosphate (S1P) lyase knockout in bone marrow-derived cells led to local sphingolipid accumulation, causing CAC development [182] via IL-23 STAT-3 signaling [183,184,185]. However, in cancer-induced inflammation during tumor development, S1P2 and EGFR signaling stimulated T-helper 2 and production of IL-23 by immune cells in the tumor microenvironment [182]. The critical finding was that inflammation-induced cancer and cancer-induced inflammation are developed by different pathogenetic steps [182], which can be essential for understanding the molecular mechanisms of cannabinoid anticancer effects and reveal more pathogenetic links in inflammation-induced CRCs.

Understanding the mechanistic links in the development of different molecular subtypes of CRC is crucial for the development of the most effective treatments. As we already discussed, analysis of changes in cannabinoid levels and other ECS components may not only shed light on how different CRCs develop, but also allow the identification of highly effective medications for the prevention and treatment of CRCs, especially CACs.

## 5. Changes of the Endocannabinoid System in CRC

In this section, we are going to discuss changes of the ECS in precancer lesions and CRC development. This may help in understanding the role of ECS alterations in CRCs and reveal the molecular mechanisms of its preventive and therapeutic effects on intestinal diseases.

### 5.1. Changes in Cannabinoid Receptors

Intact enterocytes express CB1 receptors; however, during intestinal inflammation or carcinogenesis, levels of CB1 receptors progressively decrease due to CpG island hypermethylation of the *CB1R* promoter’s transcription sites [90]. In contrast, CB2 receptors’ expression increases, which in some cases is associated with poor prognosis of CRC patients [90]. The experiments performed Wang et al. (2009) on 10 CRC cell lines (HCT-116, HT-29, LS-174T, SW-480, Colo-201, DLD-1, Caco-2, HCT-15, HCA-7, LoVo) showed that HCT-116, HT-29, and LS-174T have low CB1 expression. In the SW-480 cell line or normal colon tissues, there was no CB1 receptor change. Additionally, CB1 receptor loss was indicated in 8 out of 13 human tumor samples, showing that in human biopsies of the second and third grade of CRC, there was loss of expression of CB1 receptors often via CpG island promoter hypermethylation [16]. The transcriptome analysis of 566 CRC patients showed reduction of CB1 expression in the TNM-I stage; however, with the disease’s progression, CB1 expression was again elevated. In contrast, GPR55 expression decreased with disease progression, compared to healthy colon samples [36]. Moreover, *CpG* methylation of the *CB1* promoter site was hypomethylated in the majority of healthy intestinal tissues and CRC patients—a cohort of 86 [36]. These findings suggest that translational regulation of miRNAs may be the critical point of CB1 receptor expression changes in CRC patients [36]. Additionally, CB1-deficient APC-mutated mice had 2.5–3.8-fold elevated polyp formation, compared to the control mice [16]. However, the deletion of CB2 receptors did not significantly affect the formation of intestinal preneoplastic lesions in animal models [16]. The DLD-1 CRC xenograft model had higher CB2 levels of expression, and treatment with CB2 agonist such as *N*-cyclopentyl-7-methyl-1-(2-morpholin-4-ylethyl)-1,8-naphthyridin-4(1*H*)-on-3-carboxamide (CB13) significantly decreased tumors in size [12].

The prognostic value of the cannabinoid system in CRCs was proposed by Zhang et al. (2017), who detected the methylation status of 2245 *CB1R* promoter regions in peripheral blood samples taken from patients suffering from colon adenocarcinomas and adenomas, as well as healthy individuals [186]. Comparing CB1 promoter epigenetic changes between groups strongly suggested that CRC progression is positively associated with *CB1R* methylation. Moreover, the methylation at the 2245 locus significantly correlated with tumor size, depth of invasion, tumor stage, and lymphatic node metastases. Thus, it may be used as a prognostic marker and a screening tool for CRCs [186]. In another study, scientists analyzed 534 CRC patients’ immunohistochemistry samples, which showed the CB1 receptor to be expressed in 76.6%. Low CB1 expression (<66%) was often identified in patients with stage IV CRC. At this stage, low CB1 levels were associated with poorer prognosis. Despite the tumor grade, there were no significant differences between patients’ age, gender, histological differentiation, and tumor site between high and low CB1 expression [187].

The study provided by Tutino et al. (2019), which involved samples from 59 CRC patients, showed low expression of CB1 receptors in primary tumors and adjacent mucosa, especially in those with diagnosed metastatic disease. In metastatic CRC, downregulation of CB1 expression was associated with decreased p38/MAPK and ERK1/2 signaling in both tumor tissue and adjacent normal mucosa. Additionally, there was significant upregulation of prosurvival AKT pathway in the same samples, especially in the surrounding tumor tissues that were intact. Moreover, patients with metastatic disease had significantly lower levels of Bcl-2-associated-X protein (BAX), which is responsible for the stimulation of apoptosis [17].

Some studies showed that CB receptor expression changes play a procancer role in colon carcinogenesis [188,189,190]. Analyses of CRC samples (both tumor front and interior) for CB1 expression from 487 patients that underwent surgical resection showed that the levels of CB1 presence is associated with tumor grade. Additionally, the authors suggested that high CB1 expression is associated with poorer prognosis in stage II microsatellite stable tumors [189]. The data comparison set were variables including gender, tumor site, radiotherapy, stage, tumor differentiation, type, microsatellite stability, lymphocyte infiltration, and frequency of tumor aggregates at the invasion front. Surprisingly, the significant differences associated with CB1 receptor expression were histological tumor grade and microsatellite stability. In both tumor front and center samples, the CB1 receptor expression was higher in patients with moderate-poorly/poorly differentiated microsatellite-stable CRCs [189]. One of the explanations for the relationship between poor patients prognosis and high CB1 expression was proposed by studies on glioblastoma cell lines [190]. This study suggested that at low levels of expression, CB receptors are mainly coupled with ERK, thus, their activation caused apoptosis. It was also shown that high CB1 expression could cause additional AKT signaling activation that switches proapoptotic signaling to survival mechanisms. Conclusively, endocannabinoid levels could protect intestinal mucosa from damage; however, they may also exacerbate cancer cell survival [189]. Thus, alterations in CB receptor expression can be used as a prognostic marker in different molecular subtypes of CRC.

Recent data provided by Hasenoehrl et al. (2018) have shown that cannabinoid receptor GPR55 activation has pro-cancer effects by stimulating tumor invasiveness and promoting metastatic potential [36]. Moreover, there is evidence that blocking GPR55 with CBD activates MAPK/p53 signaling and stimulate ERK1/2, which can cause apoptotic cell death [191]. GPR55 is a G*α*12/13 and Gq lysophosphatidyinositol type of receptor, stimulating proliferation, invasion, and angiogenesis of cancer cells [192,193]. GPR55 can activate a cascade of reactions involving calcium mobilization [194], ERK1/2 phosphorylation [195], adhesion, and migration of colon cancer cells that may lead to liver metastasis [196]. Except for cancer cells, GPR55 is expressed by macrophages, neutrophils, and lymphocytes [192,197,198]. In the azoxymethane/dextrate sulfate sodium (AOM/DSS) animal model of CAC, activation of GPR55 caused changes in myeloid-derived suppressor cells and T lymphocytes that are usually present within the tumor’s microenvironment. GPR55 can increase proinflammatory molecules such as COX-2 and STAT3, which can assist in tumor initiation and progression. It was shown that GPR55 has the opposite effect to the CB1 receptor, which is considered to be protective against colonic inflammation. Knocking down the GPR55 receptors in mice models increased the levels of CD4+ and CD8+ cells in the tumor beds, which emphasizes the role of GPR55 in inflammation-induced colon cancers [36]. The study presented by Hasenoehrl et al. (2018) showed that GPR55-/- mice had decreased tumor burden when compared to the wild type [36]. In the experiment using the GPR55-/- colitis-associated colon cancer mice model, the levels of COX-2, STAT3, thromboxane A2, PGF2*α* and myeloid cell-recruiting chemokine monocyte chemoattractant protein-1 were lower than in the wild-type mice. As opposed to cytokines IL-5, IL-10, and IL-12, which were elevated in the GPR55-/- model [36]. As opposed to GPR55-/-, the CB1-/- knockout mice developed a higher number of tumors, with larger areas in different CRC models, i.e., spontaneous tumor progression and CAC. Additionally, the colon’s nontumor parts exposed to AOM/DSS had higher expressions of CB1 compared to the healthy nonexposed ones. GPR55 mRNA levels were decreased in nontumor exposed tissues and elevated in tumor lesions, compared to healthy controls [36]. Additionally, provided analysis of the available CRC patient dataset, it was revealed that patients with increased GPR55 expression have shorter relapse-free survival [36]. These results show the importance of increased expression of GPR55 in the tumors’ microenvironment regulation, inflammation, and cancer progression.

The research performed by Raup-Konsavage et al. (2018) showed the sensitivity of different molecular subtypes of CRC cell lines (SW480, SW620, HT-29, DLD-1, HCT-116, LS-174T, RKO) to 370 different cannabinoid compounds [15]. It was found that cell lines SW480, SW620, HT-29, and DLD-1 had APC mutations and HCT-116 and LS-174T had activating mutations in CTNNB1, the *β*-catenin encoding gene. SW620 is derived from lymph metastases. The identified synthetic cannabinoids that suppressed CRC cell viability most effectively were—HU-331; CP 55,940; 5-epi-CP 55,940; CP 47,497; 3-epi-CP 47,497 C-8 Homolog; CP 47,497 C-8 Homolog; PTI-1; PTI2; and NPB-22. The selected compounds did not work through canonical signaling, including CB1, CB2, GPR55, and TRPV1 [15]. The most significant result was that the cell lines with APC mutations (SW480, HT-29, DLD-1). They were more sensitive to CBD than the cells mutated in the *β*-catenin pathway (HCT-116, LS-174T) [15]. These results suggest that various molecular subtypes of CRC may react differently to cannabinoid treatment and that the antitumor action of cannabinoid compounds is not always CB1- or GPR55-dependent.

### 5.2. Changes in the Level of Endocannabinoids

**Figure 2:**
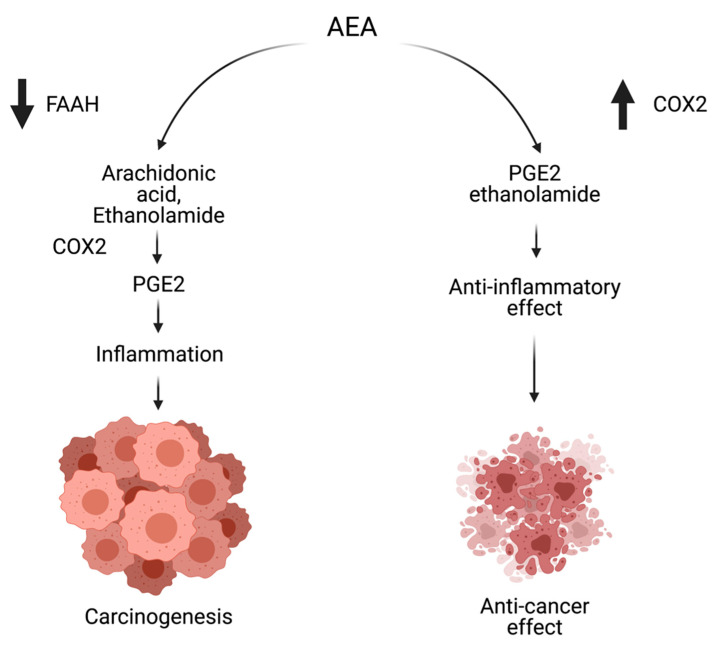
The protective role of anandamide in high COX-2 expressing colorectal cancers. See text for discussion. Created with BioRender.com (accessed on 10 August 2021). Abbreviations: AEA—anandamide; COX-2—cyclooxygenase 2; PGE2—prostaglandin E2.

Ligresti et al. (2013) performed one of the first crucial studies emphasizing the endocannabinoid system’s role in colon tumors [18]. The authors were the first to demonstrate that in CRC, 2-AG and AEA are 2–3-fold higher than in normal mucosa, but in adenomas, endocannabinoids are more elevated than in carcinomas [18,199]. Additionally, as a result of FAAH upregulation, there was an increase in arachidonic acid levels, contributing to CRC-induced inflammatory responses [199]. Other cannabinoid enzymes such as NAPE-PLD and MAGL were also elevated in CRC specimens [83,200] (see Figure 2). In the AOM mouse model, which induced ACF formation in the intestines, there was increased 2-AG expression in the colon, and treatment with FAAH inhibitor AA-5-HT decreased the formation of ACFs through an increase of AEA and 2-AG levels [32,134]. In one of the studies performed on Caco-2 cells, which are capable of differentiating in the cell culture into a low-malignant non-invasive enterocytes [201], it was shown that the inhibition of FAAH in these cells elevate levels of endocannabinoids and decrease cell proliferation [18]. However, when Caco-2 cells differentiate into non-invasive cell types, the endocannabinoid levels were reduced, FAAH increased, and the cells did not respond to cannabinoid agonists [18]. Moreover, levels of CB1 receptor expression are approximately the same in differentiated versus undifferentiated Caco-2 cells, but in differentiated cells, the native form of the CB1 receptor is higher than in undifferentiated [18], which indicates a protective role of native forms of CB1 receptors on the intestinal mucosa.

Preneoplastic intestinal lesions such as ACF induced by AOM in mice are associated with elevated endocannabinoid 2-AG and decreased cleaved caspase 3 and caspase 9. Izzo et al. (2008) provided evidence that inhibition of FAAH by N-arachidonoyl serotonin increased levels of endocannabinoids and, as a result, completely prevented ACF formation as well as normalized the levels of caspase 3. This effect was independent of CB receptor expression, suggesting that other receptor signaling may be involved, possibly TRVP1 [32].

Most of the CRCs have a higher expression of COX-2 that converts arachidonic acid into inflammatory mediator PGE2, promoting colon tumorigenesis via the activation of angiogenesis, immune response, and stimulation of cell proliferation [202,203,204,205]. Because the endocannabinoids AEA and 2-AG can be the substrates for COX-2, there is a lower level of arachidonic acid to be used to form PGE2. Thus, the anticancer effects of these molecules can be linked to inhibition of proinflammatory PGs formation [18]. This hypothesis was supported by the very low levels of COX-2 in differentiated Caco-2 and DLD-1 cells [18]. Another study performed by Kozak et al. (2002), which linked anticancer effects of cannabinoids to inflammation, showed that AEA—which can be converted by COX-2 to prostaglandin-ethanolamides—inhibits the growth of CRC cell lines that highly express COX-2 (HT-29, HCA7/C29) [206]. However, AEA had little effect on SW-480—which contains low levels of COX-2 expression. Moreover, inhibition of FAAH potentiated the cytotoxic effect of AEA on COX-2-expressing CRC cell lines [207]. These results indicate the potential preventive role of endocannabinoids against colonic tumor development, especially in inflammatory-induced CRC.

Overall, the ECS can change dynamically in both CAC- and CRC-induced inflammation, but it is still not proven whether it is protective in both cases. Summarizing our literature analysis regarding CRC, cannabinoid system, and inflammation, we can say that inflammation-induced cancer and cancer-induced inflammation have different pathogenetic development mechanisms. ECS changes are seen in both cases. However, endocannabinoids’ protective effect is more prominent in CAC models, emphasizing the role of cannabis in preventing inflammation-induced colon cancers. In contrast, higher CB1 receptor expression correlated with poorly differentiated microsatellite-stable CRCs [189]. Microsatellite-stable cancers usually have less immune cell infiltrates due to lower production of neoantigens compared to high-MSI CRCs [189]. The neoantigens produced by cancer cells may activate immune T-cell response against CRC. The presence of T-helper 1 cells stimulates cytotoxic T-cells, causing tumor growth inhibition and suppression of metastatic invasion [208]. However, in microsatellite-stable CRCs, the higher expression of CB1 receptors may contribute to immune cell evasion via the immunosuppressive effects of ECS due to a shift of T-helper 1 to T-helper 2 response [132]. Thus, in further research regarding cannabinoids, it is essential to carefully choose cell lines and animal models of CRC, especially considering the main initial pathogenic processes that drive the development of particular intestinal tumors.

## 6. Molecular Mechanisms of Anti-CRC Effects of Cannabinoids

Over recent years, multiple experimental data have provided evidence of the antioncogenic impact of cannabinoids on CRC [12,26,28,188,199,209]. Phytocannabinoids reduce CRC cell growth by multiple mechanisms of action [12,26,28,83,210], which are discussed in this section.

### 6.1. Ceramide

Ceramide is a neutral lipid backbone of complex sphingolipids. Its de novo synthesis can be activated by chemotherapy, ionizing radiation, and enzyme sphingomyelinase. Ceramide action is specific to its carbon chain lengths [211,212]. Ceramide can trigger apoptosis and inhibit cancer cell proliferation by causing cell cycle arrest. It can also activate autophagy in cancer cells. The main pathways in ceramide interaction are protein phosphatase 2A, p38/MAPK, JNK, AKT, protein kinase C, and survivin [213]. Some cancers upregulate ceramide-degrading enzymes to avoid death or even promote mutagenicity [213].

**Figure 3:**
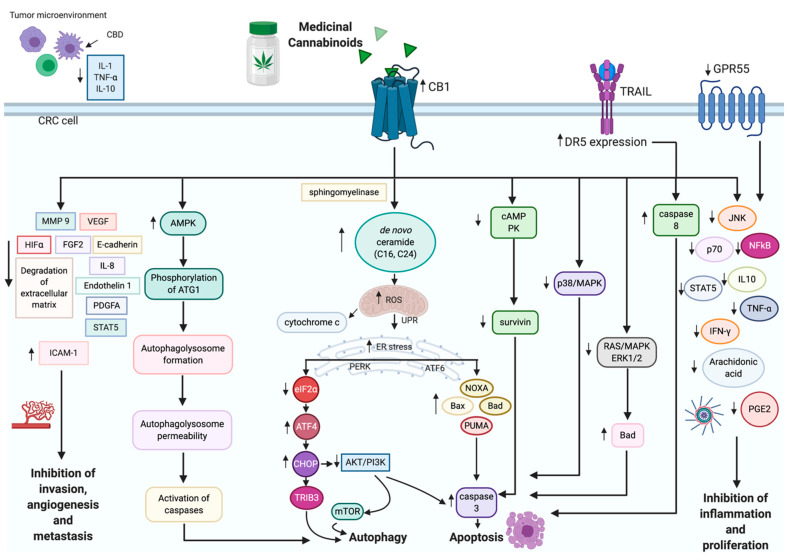
The effects of cannabinoids on CRC. See text for discussion. Created with BioRender.com (accessed on 10 August 2021). Abbreviations: AMPK—AMP kinase; ATF4—activated transcription factor 4; AKT—protein kinase B; CBR—cannabinoid receptor; cdc2—cell division control 2; cAMP PK—cyclic AMP protein kinase; CBD—cannabidiol; CHOP—C/EBP homologous protein; DR5—death receptor 5; eIF2*α*—eukaryotic initiation factor 2*α*; ERK1/2—extracellular regulated kinase 1/2; FGF2—fibroblast growth factor 2; GPR55—G protein coupled receptor 55; HIF*α*—hypoxia inducible factor *α*; ICAM-1—intercellular adhesion molecule 1; IFN-*γ*—interferon *γ*; IL-1—interleukin 1; IL-8—interleukin 8; IL-10—interleukin 10; MAPK—mitogen-activated protein kinase; MMP 9—matrix metalloproteinase 9; STAT-5—signal transducer and activator of transcription 5; mTOR—mammalian target of rapamycin; PDGFA—platelet-derived growth factor A; PGE2—prostaglandin 2; PI3K—phosphoinositide-3 kinase; TNF-*α*—tumor necrosis factor *α*; TRAIL—Tumor necrosis factor-related apoptosis-inducing ligand; TRIB3—tribbles homolog 3; VEGF—vascular endothelial growth factor.

One of the best-explained anticancer mechanisms of cannabinoids is the activation of the de novo synthesis of ceramide via CB receptor activation (see Figure 3) [12,214]. Due to the intensive synthesis of ceramide, the production of ROS is enhanced, leading to ER stress response. Next, ER stress-related signaling events may cause CRC cell death. First, eukaryotic translation initiation factor 2*α* (eIF2*α*) is downregulated, and the global translation of proteins is decreased. Simultaneously, the C/EBP homology protein (CHOP) is activated; this protein acts on pseudokinase tribbles-homologue 3 (TRIB3), which stimulates the release of proapoptotic BAD and BAX proteins [215]. Moreover, AKT is downregulated by CHOP. AKT inhibition causes major intracellular changes, such as the downregulation of the mammalian target of rapamycin (mTOR) and the activation of autophagy. In addition, AKT can directly activate caspase 3 and caspase 9 and stimulate G1 cell cycle arrest through cyclin-dependent kinase inhibitors, such as p21 and p27. The anti-tumor mechanism of cannabinoids also involves the upregulation of AMP protein kinase (AMPK), which, along with low mTOR, strongly stimulates the macroautophagy of CRC cells [12,16,26,27,191,201,216].

Cianchi et al. (2008) performed a study regarding CB receptor expression in human specimens (24 samples of primary sporadic adenocarcinoma and adjacent tissues), DLD-1, and HT-29 CRC cell lines, which showed CB1 expression mainly in normal colonic epithelial tissue samples [12]. In addition, the tumor tissues highly expressed CB2 receptors. The activation of CB1 by the synthetic agonist arachinodyl-2′-chloroethylamide and the CB2 agonist CB13 caused the stimulation of apoptosis by de novo ceramide synthesis in intestinal cancer cells. This study also showed that TNF-*α* connects CB receptor activation and ceramide synthesis in CRC cell lines, which activate apoptosis. CB1 receptor activation elevates intracellular ceramide levels via sphingomyelin hydrolysis by coupling with factors associated with neutral sphingomyelinase activation that bind to TNF receptors, resulting in sphingomyelin breakdown and ceramide de novo production [12,217]. This signaling is antiproliferative and proapoptotic to CRCs [12].

Chen et al. investigated the connection between cannabinoid signaling and ceramide, and described the profiles of the main endocannabinoids AEA and 2-AG, ceramides, free fatty acids, and the critical enzymes of cannabinoid metabolism in 47 pairs of human CRC samples and adjacent non-cancerous tissues [199]. Results showed that AEA and its metabolite, arachidonic acid, are elevated in CRC tissues and are mainly associated with lymphatic node metastases. In the CRC samples, the ceramide levels have different expression patterns, with elevated C16 and C24 and decreased C18 and C20. In addition, the mRNA levels of NAPE-PLD, FAAH, and ceramide synthases, such as CerS2, CerS5, and CerS6, are higher in cancer tissues [199]. Other experiments have shown that C16 and C24 ceramides promote apoptosis of CRC cells [218,219]. In summary, elevated levels of AEA, ceramides, and CB1 receptors are shown to have a protective effect against colon carcinogenesis [198].

### 6.2. Apoptosis

In CRCs, the RAS-MAPK pathway is overactivated, with KRAS and BRAF being overexpressed in approximately 50% and 15% of cases [220,221]. PI3K/AKT signaling is upregulated in almost 40% of colon malignancies [222]. In 2007, Greenhough et al. (2007) reported that in vitro THC-treated adenoma (AA/C1, AN/C1, BH/C1, RG/C2, AAC1/SB/10C) and CRC (SW480, HCT-15, HT-29, Caco2, HCT-116, LS-174t, SW620, and JW2) cell lines induce apoptosis via proapoptotic BAD activation by its dephosphorylation on serine 112 and 136 [26]. These effects are achieved by inhibiting the major cancer survival pathways—RAS/MAPK, ERK1/2, and PI3K/AKT via CB1 receptor activation [26]. However, THC does not affect p38/MAPK and JNK signaling [26]. The provided data showed that the induction of CB1, but not CB2 receptors, by THC can result in CRC cell death [26]. On the contrary, glioblastoma and lung carcinoma cell line treatment with nanomolar concentrations of THC may even promote cancer cell growth, which depends on metalloproteinase and EGFR activity. EGFR receptor signaling is the mechanistic link with cannabinoid receptors and can activate the pro-survival AKT pathway via the shedding of pro-amphiregulin and pro-heparin-binding epidermal growth factor-like growth factor by the TNF-*α* converting enzyme TACE/ADAM17. The experimental results showed that THC leads to the phosphorylation of EGFR, the phosphorylation of adaptor protein Src homology 2 domain-containing, and the subsequent activation of ERK1/2 and AKT/PKB pathways. EGFR transactivation with CB1/2 receptors requires EGFR tyrosine kinase and metalloproteinase activity. As a result, higher THC concentrations can induce apoptosis in multiple cell lines, although THC can accelerate cancer cell progression in nanomolar concentrations [223].

CBD is a partial agonist of CB1 and CB2 receptors [224,225]; it stimulates TRPV1, TRVP2, 5-HT1A, and PPAR *γ*, inhibits GPR55, and increases endogenous AEA concentration by blocking its hydrolysis [226]. One of the best described anticancer effects of CBD is the activation of NOXA, suppressing mTOR/AKT signaling and MAPK [28]. Recent studies performed on HCT-116 and DLD-1 CRC cell lines indicated that CBD can induce apoptosis via the significant upregulation of NOXA-ROS signaling [28]. ROS can induce ER stress response by triggering unfolded protein response (UPR) [227,228]. The ER stress can stimulate UPR to restore protein homeostasis. UPR is guided by the signaling proteins inositol-requiring protein-1*α* (IRE1*α*), protein kinase RNA-like ER kinase (PERK), and activating transcription factor 6 (ATF6). Usually, PERK and ATF6 are kept inactive by binding to the binding immunoglobulin protein (BIP) chaperone. IRE1*α* is activated directly under unfolded proteins and then starts to accumulate. When UPR is activated, all three proteins are signaled to increase the levels of chaperones, decrease translation, and transport misfolded proteins back into the cytosol for ubiquitination and subsequent degradation [228]. In CBD-treated CRC cells, the expression of the stress-related ER gene is decreased, with further activation of NOXA [28]. Activating transcription factor 3 (ATF3) and ATF4 may be involved in CHOP and NOXA stimulation [229]. The addition of CBD stimulates ER response, which results in ATF3 and ATF4 binding directly to ATF/cAMP response element in the promoter region of NOXA and CHOP [28]. As a result, NOXA migration into mitochondria causes the release of cytochrome c; the further activation of caspase 3, caspase 8, and caspase 9; the cleavage of PARP; and the initiation of apoptosis in CRC in vivo and in vitro models [28]. In addition, CBD may enhance the phosphorylation of p38 stress protein kinase, which eventually leads to apoptosis [210].

The combination of CBD with TNF-related inducing apoptosis ligand (TRAIL) causes a synergistic effect of the two molecules on CRC in vivo. CBD treatment activates ER stress response with CHOP release and phosphorylated protein kinase RNA-like ER kinase (PERK). In addition, CBD stimulates the expression of molecules responsible for the extrinsic apoptotic pathway by stimulating DR5 expression. The addition of 4 μM CBD to 10 ng/mL TRAIL potentiates the effect of TRAIL by sensitizing CRC cells to undergo TRAIL-induced apoptosis in a xenograft mouse model [230]. Moreover, CBD suppresses the expression of the inhibitors of apoptosis (IAPs) c-FLIP and survivin [230]. IAPs are a group of antiapoptotic molecules that suppress caspase activity [231]. One of the IAPs, survivin, is overexpressed in merely every tested tumor and may serve as a promising target molecule for CRC treatment [232]. CBD may exhibit a protective role against CRC by the stimulation of CB1 receptors, which causes the inhibition of cAMP-dependent protein kinase. This process leads to the reduction of the cdc2 (Wee1/cdc25C-cdc2 cascade), which leads to the destabilization of survivin, the activation of caspase 3, and apoptosis [16]. These findings show that cannabinoids can become efficient preventive agents in canonical molecular subtypes of CRCs [16].

Another phytocannabinoid, cannabigerol (CBG), a partial agonist of CB1 and CB2 receptors [233], inhibits endocannabinoid reuptake [33]. CBG is also a potent HT5_1A_ antagonist [233]; an agonist of TRPA1, TRPA2, and TRPV2; and an antagonist of TRPM8 [33]. CBG has chemopreventive effects in AOM-induced colon carcinogenesis via the activation of apoptosis, the stimulation of free radical formation, the upregulation of CHOP, and the inhibition of CRC cell growth. These effects are enhanced by CB2 antagonists and TRPM8 agonists [234].

### 6.3. Extracellular Vesicles

Extracellular vesicles are classified into exosomes, microvesicles, and apoptotic bodies [235]. Exosomes and microvesicles mediate intercellular communications by carrying molecules from parental cells to recipient cells. These lipid-bilayer vesicles can affect the physiology of migration, differentiation, and angiogenesis of cancer cells [236,237,238]. Vesicular release is regulated by membrane receptors, apoptotic signals, and intracellular calcium release [239]. It was shown that apoptotic bodies can transfer oncogenes horizontally, resulting in cancer cell survival [240]. Thus, tumor-derived exosomes prepare a pre-metastatic niche in specific organs [241]. Kosgodage et al. (2018) showed that CBD can inhibit cancer-derived extracellular vesicles release in a dose-dependent fashion. The effect is associated with alterations in mitochondrial functions, the modulation of STAT3 signaling, and changes in prohibitin expression [242]. As a result, CBD may sensitize cancer cells to chemotherapy drugs via the alteration of the biogenesis of extracellular vesicles [242].

### 6.4. Autophagy

Autophagy is the self-consumption mechanism that eliminates intracellular waste, attenuates stressful factors, and exhibits anti-carcinogenic effects [243]. One of the conventional chemotherapy drugs used in CRCs is oxaliplatin, which causes the formation of DNA crosslinks, usually between guanines and guanine-adenine, effectively killing cancer cells [244]. However, 40% of patients with CRC may develop resistance to it [245]. Jeong et al. (2019) showed that CBD can overcome oxaliplatin resistance through the activation of autophagy and the inhibition of superoxide dismutase 2, a main antioxidant enzyme within a cell [246]. Moreover, the authors observed decreased phosphorylation of nitric oxide synthase 3 (NOS3), resulting in reduced NO and ROS production [246]. The study suggested that NOS3 phosphorylation is indispensable for the development of oxaliplatin resistance. Oxaliplatin resistance can be overcome by the addition of CBD to the treatment, which results in autophagy-mediated cell death and the formation of free radicals by dysfunctional mitochondria in resistant cells [246]. Combined CBD treatment with oxaliplatin causes the activation of the microtubule-associated protein 1A/1B light chain 3B and the increased expression of p63 [246]. These markers are commonly used to assess autophagy [247]. In addition, the combination of CBD and oxaliplatin decreases the number of mitochondria in the resistant cells and reduces the levels of cardiolipin and NADH dehydrogenase 1*α* subcomplex subunit 9 (mitochondrial complex I), resulting in abnormal oxidative phosphorylation and autophagy-mediated cancer cell death [246]. Other studies regarding drug resistance and cannabinoids indicated that THC, CBD, and CBN can inhibit ATP binding cassette family transporters, P-glycoprotein, and the breast cancer resistance protein BCRP [248], resulting in the potential chemosensitizing effect of cannabinoids in resistant CRCs [249,250,251].

### 6.5. CRC Angiogenesis and Metastasis

Cannabinoids can inhibit the invasion and metastasis of cancer cells by downregulating vascular endothelial growth factor (VEGF), matrix metalloproteinase 2 (MMP2), MMP9, and the adhesion molecule E-cadherin [252]. In a xenograft and AOM model of colon carcinogenesis, Pagano et al. (2017) showed that 2-AG and MAGL, a serine hydrolase that degrades 2-AG, are highly expressed in aggressive colon cancers. The inhibition of MAGL by URB602 decreases xenograft tumor volume through the downregulation of VEGF and fibroblast growth factor-2 (FGF-2). This study has shown that 2-AG exerts an anti-tumor effect in colon cancer via the inhibition of angiogenesis (VEGF) and cell proliferation (cyclin D1). Moreover, in a mouse AOM model of colon carcinogenesis, URB602 attenuates the formation of preneoplastic lesions, such as polyps, thus supporting the chemopreventive role of endocannabinoid 2-AG in CRC [252]. The in vitro experiments provide evidence that 17*β*-estradiol stimulates CB1 expression via the activation of the estrogen receptors ER*α* and ER*β* in the primary tumor CRC cell lines DLD-1 and HT-29 and the lymph node metastatic cell line SW620. The authors suggest the antiproliferative effect of estrogens on primary and metastatic CRCs via interaction with polyamine and growth factors in the tumor [134,253].

In another study, the strong antiangiogenic effect of the cannabinoid-like compound LYR-8 was demonstrated on a xenograft model using chick chorioallantoic membranes. The mechanism behind cannabinoid action is the suppression of VEGF, COX-2, and hypoxia-inducible factor *α* (HIF*α*) [254]. One of the synthetic cannabinoids, HU-311, which is a quinone of CBD, shows antiangiogenic effects by stimulating apoptosis in endothelial cells and inhibiting topoisomerase II [255]. Some experiments have also demonstrated that 12 μM CBD may induce the migration of HUVECs. CBD inhibits MMP 2, MMP 9, and tissue inhibitor of metalloproteinase 1, which results in the suppression of cell motility and the invasion of endothelial cells; also, CBD inhibits urokinase-type plasminogen activator (uPA) and serpin E1/plasminogen activator inhibitor 1, which are involved in the degradation of the extracellular matrix and contribute to cancer cell invasiveness. Moreover, CBD downregulates HIF1*α* in U87 cells, which suggests the suppression of cell survival, motility, and angiogenesis [256]; endothelin 1, PDGF-A [257]; and the reduction of STAT5-induced vasorelaxation [256,258]. In addition, the antimetastatic action of CB receptor agonists have been shown on SW480 cell lines. AEA, HU-210 (non-selective CB agonists), and docosatetraenoylethanolamide (CB1 selective agonist) suppress the norepinephrine-induced migration of human CRC cells [134,259].

### 6.6. Irinotecan and THC

Prester et al. (2018) suggested that the combination of the chemotherapy drug irinotecan with THC potentiates the toxic effects of chemotherapy. Irinotecan, the topoisomerase I inhibitor, is one of the most commonly prescribed chemotherapy for metastatic CRC [260]. In this study, rats were injected intraperitoneally with irinotecan and subsequently THC, which resulted in more prominent leukopenia than irinotecan injections alone. This study shows that THC cannot alleviate irinotecan’s side effects, including leukopenia and diarrhea [260]. However, further experiments are needed to investigate the potential combinational value of cannabinoids and topoisomerase inhibitors. Moreover, treatment with THC decreases the levels of aspartate aminotransferase, highlighting its hepatoprotective role. Notably, cannabinoids are easily bound to plasma lipoproteins; consequently, they can interact with protein-bound drugs, including chemotherapeutics [260], which may alter the effects of conventional chemotherapy.

### 6.7. Cannabis Extracts over Purified Cannabinoids

Experiments on the CRC cell lines DLD-1 and HCT-116 indicate the significant inhibition of proliferation by high-CBD *Cannabis sativa* extracts [29]. These studies also indicate a higher affinity of CBD-extract to CB1 and CB2 receptors compared with purified CBD. In addition, the same extract decreases polyp formation in an AOM animal model and reduces neoplastic growth in xenograft tumor models [29].

Nallathambi et al. (2017) showed synergistic interaction within different fractions of *C. sativa* extract that results in colon cancer G0/G1 cell cycle arrest and apoptosis [34]. This study shows that the extracts high in cannabigerolic acid (CBGA), and THCA exerts the most potent anticancer effect. THCA shows immunomodulatory, anti-inflammatory, and antineoplastic activity, whereas CBGA has predominantly cytotoxic activity. The suppressed expression of genes, such as cyclin E2 and cyclin E1, causes cell cycle arrest. In addition, TRAIL and PUMA genes are stimulated under the combination of extracts, which results in the apoptosis of CRC cells [34].

On the contrary, Raup-Konsavage et al. (2020) indicated that full spectrum CBD oils did not reduce cell viability of CRC, melanoma, and glioblastoma cell lines more effectively than pure CBD. In fact, purified CBD showed lower IC_50_ concentrations than CBD oils [261].

The controversial data regarding the effectiveness of cannabinoids vs. cannabinoid extracts show that the variabilities in concentrations of cannabinoids, terpenes, and other molecules present in cannabis plant may impact their therapeutic effects. Moreover, often the levels of these active ingredients may vary within the same strain and be influenced by growth conditions [262]. The solution could be to test the combinations of pure cannabinoids, terpenes, or other molecules of interest in known doses and on specific molecular subtypes of CRC.

## 7. Conclusions

For many centuries, the *Cannabis* plant had been cultivated and used for agricultural and medicinal purposes [263]. One of the first discovered cannabinoids is THC, which is known for its psychotropic activities. In the following years, other non-psychogenic cannabinoids, such as CBN, CBD, CBC, and THCA, have become well studied because of their various clinical effects, including anti-tumor activities. However, not all tumors can respond to cannabinoid therapy in the same manner. Furthermore, the exact mechanisms remain unclear. Potential antineoplastic drug interactions and developing drug resistance should be noted [191]. Nevertheless, we can assume that cannabinoid agonists prove to be efficient in multiple experimental models, though studies on the molecular mechanisms of their action and potential drug interactions remain scarce. More experiments involving different molecular subtypes of CRC should be performed, and clinical trials are needed to reveal the full treatment potential of cannabinoids.

This review covers known data regarding the effects of cannabinoids on intestinal inflammation and CRC. These molecules may serve as a promising novel treatment option for this devastating disease. However, more in vitro and in vivo studies are required. Given that CRC is a heterogeneous disease with different genomic landscapes, experiments with cannabinoids should involve different molecular subtypes, emerging mutations, and various stages of the disease. Moreover, cannabinoid system profiles drastically change during intestinal tumor development. Hence, the choice of cannabinoids for CRC prevention and treatment can also depend on the type of CRC, its etiology (for instance, colitis-associated, familial), its driver mutations, and cannabinoid receptor expression levels. This review provides a detailed description of the molecular events that occur in CRC under cannabinoid system changes. We hope that this review can help researchers form a comprehensive understanding of cannabinoid interactions in CRCs. We believe that selecting a particular experimental in vitro and in vivo model based on the disease’s genetic landscape is a crucial step in the preclinical stages of drug discoveries.

In summary, this review aims to show that comprehension of the molecular mechanisms of the cannabinoid system is crucial for revealing new targeted treatment options, developing preventive measures, and developing novel screening and prognostic methods in chronic colon inflammation and CRC.

## Data Availability

Not applicable.

## Abbreviations

The following abbreviations are used in this manuscript:

2-AG	2-arachidonoyl glycerol
AEA	anandamide
ATF4	activated transcription factor 4
AKT	protein kinase B
AMPK	adenosine monophosphate kinase
APC mutations	adenomatous polyposis coli mutations
APC	antigen-presenting cell
cAMP PK	cyclic AMP protein kinase
CAC	colitis-associated cancer
CB (1 & 2)	cannabinoid receptor (Type 1 & 2)
CBD	cannabidiol
CBR	cannabinoid receptor
CD	Crohn’s disease
cdc2	cell division control 2
CHOP	C/EBP homologous protein
CNS	central nervous system
COX2	cyclooxygenase 2
CRC	colorectal cancer
CTC	cytotoxic T-cell
DGL	diacylglycerol lipase
DR5	death receptor 5
EGFR	epidermal growth factor receptor
eIF2*α*	eukaryotic initiation factor 2*α*
EMT	epithelial-to mesenchymal transition
ERK1/2	extracellular regulated kinase 1/2
FAAH	fatty acid amide hydrolase
FGF2	fibroblast growth factor 2
GIT	gastrointestinal tract
GPR55	G protein coupled receptor 55
HIF*α*	hypoxia inducible factor *α*
IBDs	inflammatory bowel diseases
ICAM-1	intercellular adhesion molecule 1
IFN-*γ*	interferon *γ*
IL-(1*β*, 6, 8, 10, 13, 23)	inerleukin-(1*β*, 6, 8, 10, 13, 23)
JAK	Janus kinase
MAGL	monoacylglycerol lipase
MAPK	mitogen-activated protein kinase
MMP 9	matrix metalloproteinase 9
MSI	microsatellite instability
mTOR	mammalian target of rapamycin
NAPE-PLD	N-acyl-phosphatidiyl-ethanolamine-hydrolyzing phosphatase D
OEA	oleoylethanolamide
PDGFA	platelet-derived growth factor A
PEA	palmitoylethanolamide
PGE2	prostaglandin E2
PGF2*α*	prostaglandin F2*α*
PI3K	phosphoinositide-3 kinase
PLC	phospholipase C
PPAR	peroxisome proliferator activating receptor
STAT (1, 3, 5)	signal transducer and activator of transcription (1, 3, & 5)
TH17	T-helper 17
TLR 4	Toll-like receptor 4
TNF-*α*	tumor necrosis factor *α*
TRAIL	Tumor necrosis factor-related apoptosis-inducing ligand
TRIB3	tribbles homolog 3
TXA2	thromboxane A2
UC	Ulcerative colitis
VEGF	vascular endothelial growth facto
WNT	Wingless pathway
